# Chicken Combs and Wattles as Sources of Bioactive Peptides: Optimization of Hydrolysis, Identification by LC-ESI-MS^2^ and Bioactivity Assessment

**DOI:** 10.3390/molecules25071698

**Published:** 2020-04-07

**Authors:** Taliana Bezerra, Mario Estévez, José Thalles Lacerda, Meriellen Dias, Maria Juliano, Maria Anita Mendes, Marcelo Morgano, Maria Teresa Pacheco, Marta Madruga

**Affiliations:** 1 Department of Food Engineering, Technology Centre, Federal University of Paraiba, Joao Pessoa 58051-900, Brazil; taliana.kenia@hotmail.com (T.B.); msmadruga@uol.com.br (M.M.); 2 Animal Production and Food Science Department, IPROCAR Research Institute, Avda Ciencias s/n, University of Extremadura, 10003 Cáceres, Spain; 3 Department of Biophysics Federal University of São Paulo, Rua Tres de Maio, 2 andar, São Paulo 100, Brazil; thalles_lacerda2@hotmail.com (J.T.L.); ma.juliano@unifesp.br (M.J.); 4 Dempster MS Lab-Chemical Engineering, Department of Polytechnic, School of University of Saõ Paulo, Saõ Paulo Rua do Lago 250, Bloco B 3, Saõ Paulo 05508-080, Brazil; meriellend@gmail.com (M.D.); mariaanita.mendes@gmail.com (M.A.M.); 5 Institute of Food Technology (ITAL), Brasil Ave 2880, P.O. Box 139, Campinas 13070-178, Brazil; morgano@ital.sp.gov.br (M.M.); mtb@ital.sp.gov.br (M.T.P.)

**Keywords:** protein hydrolysate, free radicals, chicken by-products, bioactive compounds, LC-ESI-MS^2^

## Abstract

The production of bioactive peptides from organic by-waste materials is in line with current trends devoted to guaranteeing environmental protection and a circular economy. The objectives of this study were i) to optimize the conditions for obtaining bioactive hydrolysates from chicken combs and wattles using Alcalase, ii) to identify the resulting peptides using LC-ESI-MS^2^ and iii) to evaluate their chelating and antioxidant activities. The hydrolysate obtained using a ratio of enzyme to substrate of 5% (*w*/*w*) and 240 min of hydrolysis showed excellent Fe^2+^ chelating and antioxidant capacities, reducing Fe^3+^ and inhibiting 2, 2′-Azino-bis(3-ethylbenz-thiazoline-6-sulfonic acid) (ABTS) and 2,2-diphenyl-1-picrylhydrazyl (DPPH) radicals. The mapping of ion distribution showed that a high degree of hydrolysis led to the production of peptides with *m*/*z* ≤ 400, suggesting low mass peptides or peptides with multiple charge precursor ions. The peptides derived from the proteins of cartilage like Collagen alpha-2(I), Collagen alpha-1(I), Collagen alpha-1(III) and elastin contributed to generation of bioactive compounds. Hydrolysates from chicken waste materials could be regarded as candidates to be used as ingredients to design processed foods with functional properties.

## 1. Introduction

The increased production of chicken meat worldwide has resulted in the production of enormous quantities of by-products generated during the slaughter process, as only the carcass has commercial value. The full exploitation of these by-products with innovative processing and industrialization technologies is of great economic importance since it adds value to the entire production chain [1]. Furthermore, global trends in sustainability and the circular economy emphasize the necessity of using the by-products from an industrial activity as raw material for another industrial activity. As a result, the accumulation (or removal) of those by-products, which involves environmental concerns and additional expenses, is avoided. By-products derived from the slaughter of chickens, including the head, skin, feathers, comb, wattle, bone, meat scraps, blood, fatty tissues, feet and internal organs, may account for 37% of the total live weight of the animal [2]. Though comb and wattle by-products are an excellent source of collagenous protein (61% dry basis), they have not been properly explored and exploited [3].

Currently, the food and pharmaceutical industries express great interest in the research and application of various transformation methods to exploit the by-products from agricultural and livestock activities [4]. One of the transformation methods applied to these by-products involves the protease-mediated hydrolysis of proteins for the generation of peptides with bioactive properties. The hydrolysis procedure needs to be carried out by controlling critical factors to meet the technical specifications of the enzyme so that the optimal hydrolytic conditions are achieved. The main factors to be controlled during hydrolysis include temperature, pH, time and enzyme/substrate ratio, and these conditions must be optimized for a specific combination of substrate and enzyme [5]. The selection of the proteolytic enzyme must be made bearing in mind the type of substrate to be degraded and the functionality of the hydrolysate to be obtained. It is consolidated in the literature that the enzyme Alcalase® from *Bacillus licheniformis* has a remarkable proteolytic potential [6]. This process results in the release of peptides with different molecular weights and specific amino acid sequences. Such peptides may display diverse bioactive and technological potential [5]. Peptides obtained from protein hydrolysates, especially those acquired from collagen hydrolysate, have received special attention from researchers due to their inherent bioactivities. A lot of literature has reported mineral chelating, antioxidant, antihypertensive, antithrombotic, antiulcerative, healing and osteoprotective properties as well as properties that improve the absorption/bioavailability of minerals [5,7].

Bioactive peptides with antioxidant activities are widely researched due to their actions in combating diseases related to oxidative stress, such as cancer, diabetes, atherosclerosis, neurodegenerative diseases, inflammatory diseases and cell aging. Antioxidant peptides have the ability to stop chain reactions, such as lipid peroxidation, by donating a pair of electrons to free radicals [8]. The ability of peptides to act as antioxidants depends on the amino acid composition and sequence, and a previous hydrolysis from the native protein is required to gain such bioactivity [9]. Sarmadi et al. [9] and Acquah et al. [10] reported the influence of amino acids with hydrophobic and/or acid character in the mechanism of the antioxidant action of peptides, as they have the ability to chelate metals and donate electrons to neutralize free radicals. Another factor of great importance for the antioxidant capacity is related to the molecular weight of the peptide chain. This bioactivity is intensified with fractions of lower molecular weight, and therefore, hydrolysis must be carried out effectively to obtain peptides from smaller fragments. Peptides of low molecular weight will have the ability to be absorbed and, thus, exert antioxidant action in organisms [11].

In view of these arguments, the objectives of this study were i) to optimize the conditions for obtaining bioactive hydrolysates from chicken combs and wattles (C + W) using Alcalase, ii) to identify resulting peptides using LC-ESI-MS^2^ and iii) to evaluate their chelating and antioxidant activities.

## 2. Results and Discussion

### 2.1. Optimization of the Hydrolysis Process

Two variables of the hydrolysis process, namely, enzyme/substrate ratio (E:S) and hydrolysis time (T) were optimized using a full factorial plan consisting of 2^2^ + 3 repetitions of the central point (7 experiments in total). The model was adjusted using Equation (1) where the degree of hydrolysis (DH) was used as response variable (R^2^ = 0.95). According to the F-test, the regression was considered significant (F_calculated_ > F_tabulated_), and there was no lack of adjustment (F_calculated_ < F_tabulated_).

DH = 13.76 + 6.59(E:S) + 5.23(T) + e(1)

**Figure 1:**
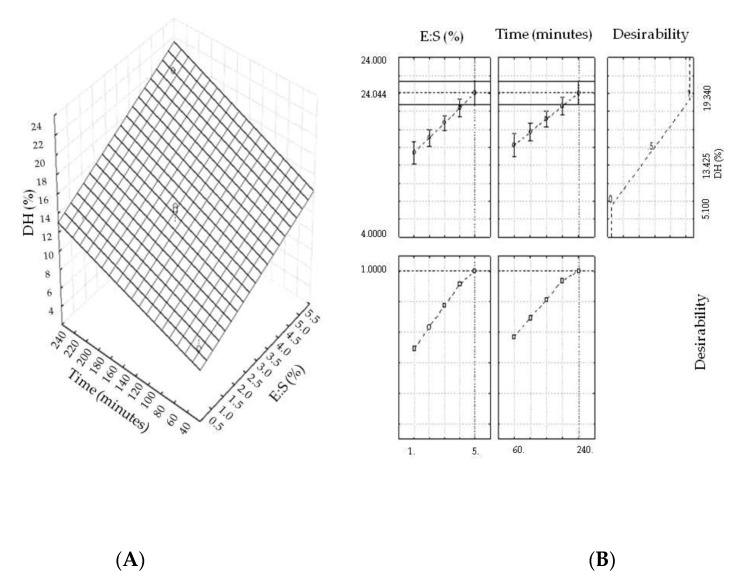
(**A**) Response surface for the dependent variable “degree of hydrolysis”. (**B**) Desirability graph of the degree of hydrolysis. DH (%), degree of hydrolysis; E:S (%), enzyme to substrate ratio; Time (minutes), hydrolysis time.

Based on the response surface (Figure 1A), the region with the best response values (>DH) was located at a hydrolysis time of 240 min and an enzyme to substrate ratio of 5% (*w*/*w*). The reaction time and quantity of enzyme (Alcalase) considered optimal for protein hydrolysis of the comb and wattle mixture was higher than that used by Chi et al. [8] for hydrolyzing fish skin (1% Alcalase, Flavourzyme and Neutrase) for 120 min. 

The desirability function was applied in this study (Figure 1B) to identify the optimized variables as indicated by the response surface. According to this study, the ranges of the enzyme to substrate (E:S) and hydrolysis time were 4.2 to 5% (*w*/*w*) and 210 to 240 min, respectively. Based on these results, the values of 5% (*w*/*w*) E:S and 240 min of hydrolysis time were considered optimal.

After selection of the optimal conditions, the model was validated, and the hydrolysis was performed in triplicate. The mean of the experimental data obtained was 18.6% ± 0.37 for the degree of hydrolysis. When this value was compared with the estimated values using ANOVA and *t*-tests (*p* ≤ 0.05), there were no significant differences. 

Thus, after optimization of the hydrolysis process, the hydrolysates with the highest (19%) and lowest (7%) degree of hydrolysis (response function) were selected for evaluation. 

### 2.2. Electrophoresis Profile 

**Figure 2:**
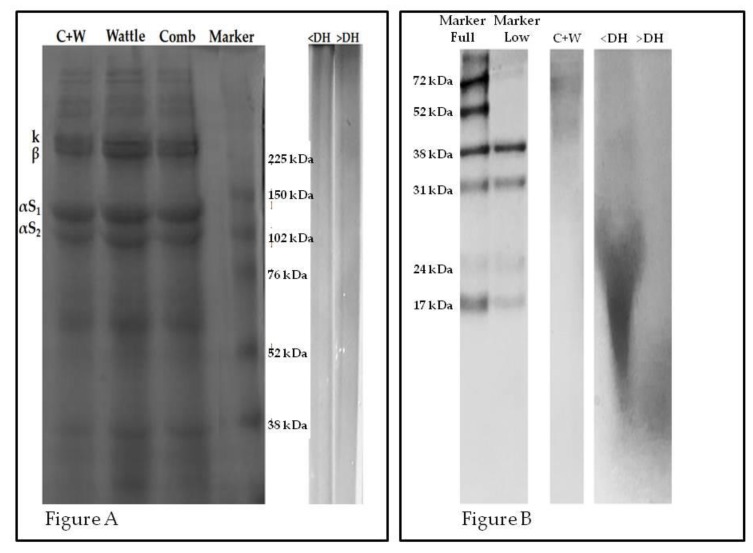
(**A**): lectrophoretic profile (SDS-PAGE) and (**B**): electrophoretic profile (SDS-PAGE- Tricine). C + W: mixture of combs and wattles; <DH: protein hydrolysate of the chicken comb and wattle mixture with the lowest degree of hydrolysis; >DH, protein hydrolysate of the chicken comb and wattle mixture with the highest degree of hydrolysis.

The profile shown in Figure 2 indicates that collagen was the protein found at the highest proportion in the raw material (C + W). The characteristic structure of collagen and a greater amount of type I can be verified by bands of higher intensities of the α, β and γ fractions [12].

The presence of two α fractions (1 and 3) resulted in two α bands, and the first band had higher intensity. The fractions of higher molecular weights represented by the β (dimer) and γ (trimer) fractions indicated collagen-rich intramolecular crosslinks. This result was similar to that found by Zhou et al. [12] in chicken feet. Other bands can be observed and may have originated from other proteins present in the raw material or may be specific to other types of collagen.

After enzymatic hydrolysis of the raw material (C + W), all of the aforementioned proteins were hydrolyzed and low molecular weight peptides were released. From the electrophoretic profile, it was observed that there were no bands of either hydrolysate in the gels, which separated proteins and peptides up to 38 kDa.

Figure 2B shows a greater intensity of low molecular weight peptides in the hydrolysate with the highest degree of hydrolysis (>DH), though the bands located in the region below 17 kDa were diffuse. By contrast, the hydrolysate with the lowest degree of hydrolysis (<DH) also showed diffuse bands in the region where peptides of intermediate molecular weight (≤24 kDa) were found, indicating incomplete hydrolysis.

### 2.3. Profile of Total and Free Amino Acids 

The total and free amino acid profile is presented in Table 1, which shows a total of 19 amino acids and highlights the remarkable concentrations of glutamic acid, aspartic acid, glycine, proline, hydroxyproline, alanine and arginine.

Glycine, hydroxyproline and proline are characteristic amino acids of collagen [13], and the results of the previous sections confirmed that collagen was the most abundant protein in the raw material. 

The bioactive properties of peptides depend mainly on their composition and sequence. While larger proportions of hydrophobic amino acids can be observed, remarkable amounts of hydrophilic amino acids such as aspartic and glutamic acid were also found. These amino acid residues can act synergistically to display relevant bioactivities. Hydrophobicity is known to influence peptide self-assembly, taste-active properties, biostability, antioxidant activity and bioavailability [10]. On the other hand, aspartic acid and glutamic acid feature chelate properties, thereby conferring antioxidant effects [9,10]. The antioxidant activity of protein hydrolysates depends on other features such as the peptide size, with this factor being discussed in due course. 

Free amino acids are the result of cleavage by the proteolytic enzyme in the region of the side chains of proteins and peptides. The higher the degree of hydrolysis, the greater the potential for protein breakdown and thus the greater the release of free peptides and/or amino acids [14]. In this study, this relation was found for all amino acids except for tryptophan and histidine, for which no significant differences were found. In addition to these bioactive properties, free amino acids are considered precursors for the formation of aromatic substances [15].

### 2.4. Fe^2+^ Chelating Capacity and Fe^3+^ Reductive Activity 

**Figure 3:**
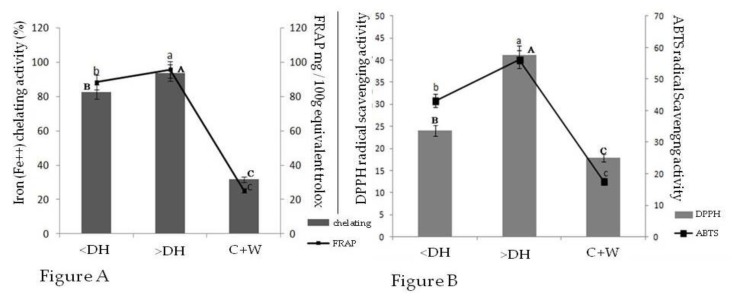
(**A**) Reducing capacity of Fe^3+^ and chelation to the Fe^2+^ metal of the chicken comb and wattle mixture (1:1 *w*/*w*) and protein hydrolysates; (**B**) ability of the chicken comb and wattle mixture (1:1 *w*/*w*) and protein hydrolysates to eliminate ABTS^●^ and DPPH^●^ radicals. C + W, chicken comb and wattle mixture; <DH, protein hydrolysate of the chicken comb and wattle mixture with the lowest degree of hydrolysis; >DH, protein hydrolysate of the chicken comb and wattle mixture with the highest degree of hydrolysis. Different uppercase letters indicate significant differences (*p* ≤0.05) between the samples regarding the chelating capacity analysis. Different lowercase letters indicate significant differences (*p* ≤ 0.05) between the samples regarding the Fe^3+^ reducing activity.

The ability of a protein hydrolysate to chelate metals with pro-oxidizing abilities, such as Fe^2+^, is considered an important indicator of potential antioxidant activity [16]. In the present study, the peptides from both hydrolysates had excellent abilities to chelate Fe^2+^. Notably, the hydrolysate with the greatest potential for protein breakdown, which contained higher amounts of low molecular weight peptides (>DH), presented a 94% iron chelating activity (Figure 3A).

The chelating potential is also related to the sizes and amino acid compositions of the peptides; the amino acids present in the peptide side chains are especially important. As reported by Zhang et al. [17], aspartic acid, glutamic acid, histidine, lysine and arginine are responsible for the greater ability of a peptide to chelate metals. The present study showed high concentrations of aspartic acid and glutamic acid. These amino acids, which confer Fe^2+^ chelating capacity and, consequently, antioxidant activity to the hydrolysates, were concluded to be present in the side chains of the peptides obtained via hydrolysis.

The reducing power of protein hydrolysates from the chicken comb and wattle mixture was measured based on the ability to donate electrons to the Fe^3+^-ferrocyanide complex to form ferrous ions (Fe^2+^). As shown in Figure 3A, the reducing capacity was proportional to the increase in protein breakdown. This potential is enhanced both by the molecular weight of the peptides released during the breakdown and by the sequence of the amino acids that compose the peptides. Elango et al. [18] associated the reductive capacity of whale shark skin hydrolysate peptides to the presence of some specific amino acids in the peptide chain, such as proline and hydrophobic amino acids.

### 2.5. Sequestering Activity for 2,2-Diphenyl-1-Picrylhydrazyl Radical (DPPH^●^) and 2,2-Azino-bis (3-Ethylbeothiazoline)-6-Sulphonic Acid (ABTS^●^) 

Based on Figure 3B, we observed that the antioxidant potential against the DPPH^●^ and ABTS^●^ radicals progressively increased with increasing amounts of protein hydrolysis. This same behavior was found by Chi et al. [7] in fish skin hydrolysates containing different fractions of varied molecular weights.

The literature reports that molecular weight is an important factor that enhances the antioxidant potential of a peptide, thus confirming the relationship between the degree of hydrolysis and an increase in antioxidant potential [14]. The hydrolysate with the highest degree of hydrolysis presented a higher concentration of low molecular weight peptides (<17 kDa); this result is described in the electrophoretic profile shown in Section 2.2. The ability of the protein hydrolysates with higher degrees of hydrolysis to inhibit the ABTS and DPPH radicals, was considered satisfactory. The determined values of the antioxidant activities in such tests were higher than those obtained by Elango et al. [18] in hydrolyzed whale shark skin collagen, and by Aléman et al. [19], in hydrolyzed squid gelatin.

### 2.6. Peptides Profile Identification from Chicken Comb/Wattle Protein Hydrolysate

In order to characterize peptides from high and low degree of hydrolysis (>DH and <DH, respectively), they were submitted to LC-ESI-MSMS analysis. In both hydrolysates, the most intense peak ions, eluted in a retention time (RT) of 20–30 min, corresponded to an acetonitrile gradient range of around 7–12%, which reveals a hydrophilic profile of the peptides. However, the mapping of the ion distribution showed that high degree hydrolysis (>DH) led to the production of peptides with *m*/*z* ≤ 400, suggesting low mass peptides, or with multiple charge precursor ions, when compared to the low degree hydrolysis (<DH). 

**Figure 4:**
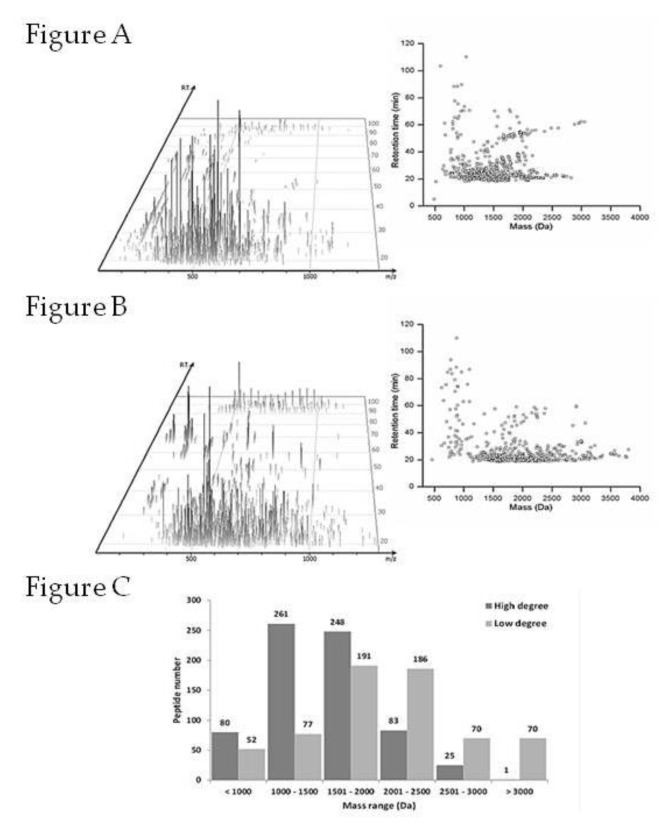
Comparison of derived peptide profiles from the different degrees of hydrolysis. 3D LC-MS showing distribution of ion peak (*m/z* × retention time × intensity) followed by characteristics of the identified peptides in the homology search according to molecular weight (MW) in high degree (**A**) and low degree (**B**) hydrolysis processes. Identified peptides number distribution according to MW range in each hydrolysate (**C)**.

The MSMS spectra submitted to a homology search resulted in 697 peptides sequences (486–3057 Da mass range) from 14 proteins at >DH and 646 sequences (461–4748 Da mass range) from 32 proteins at <DH. Although the derived peptides numbers were similar, the mass range distributions were distinct among hydrolysates. Most of the peptides from >DH hydrolysates (high hydrolysis degree) had a calculated mass <2000 Da (median 1518 Da), while those from <DH hydrolysates (low hydrolysis degree) had most peptides with a mass >2000 Da (median 2003 Da) (Figure 4). This difference could be greater, but di-tripeptides could not be identified due low spectral quality or many possibilities of coverage on proteins. 

The higher enzyme concentration leading to a high hydrolysis degree (>DH), provided small peptides and contained the highest amount of free amino acids. It is worth emphasizing that peptides with a mass lower than 3kDa have been reported as potent antioxidant agents [20,21]. Furthermore, when the most abundant peptides were compared between hydrolysates, it was observed that peptides obtained from >DH hydrolysis had an average mass of 1284 Da and a length of 13 AA, while those from <DH had a mass of 2427 Da and a length of 24 AA (Table 2). These data suggest that antioxidant activity may be attributed to a number of small peptides with characteristic sequences.

**Figure 5:**
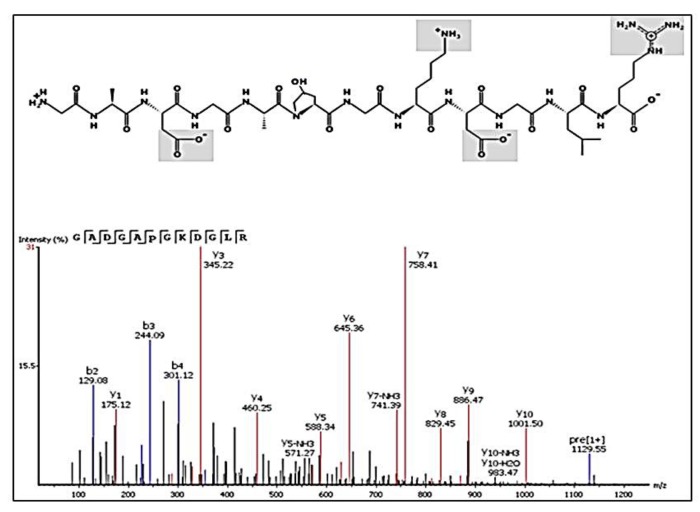
Representation of the potential antioxidant peptide based on amino acid composition. MS/MS spectrum of the peptide GADGAP (+15.99)GKDGLR (*m/z* 565.27+2) with a possible ion chelating effect through iron binding on the carboxyl oxygen of Asp, guanidine nitrogen of Arg and amino group of Lys. Post-translational modifications: hydroxylation on proline (+ 15.99 Da).

Although molecular weight is one major factor having an influence on the antioxidant ability of peptides, other structural features like amino acid composition and sequences are known to also have an impact [22]. The peptides from chicken comb/wattles hydrolysate were derived from the structural proteins of cartilage like Collagen alpha-2(I) chain, Collagen alpha-1(I) chain, Collagen alpha-1(III) chain and elastin, having mainly Gly, Pro, Val and Ala (in descending order) and a large number of their sequences with Arg, Lys, Glu, Gln, Phe and Pro in the C-terminal. Peptides containing Pro, Ala, Val and Phe were indicated as potential agents to scavenging free radicals [23], and the occurrence of Glu, Gln and Lys has also been linked to efficient abilities to chelate metal ions and scavenge hydroxyl radicals [24]. Moreover, polar/charged amino acids Asp, His, and Arg have been found to be positively correlated with iron-binding activities [25]. Thus, peptides such as GADGAP(+15.99) and GKDGLR (*m/z* 565.27^⁠+2^) found in >DH may be considered possible antioxidant agents (Figure 5). 

**Figure 6:**
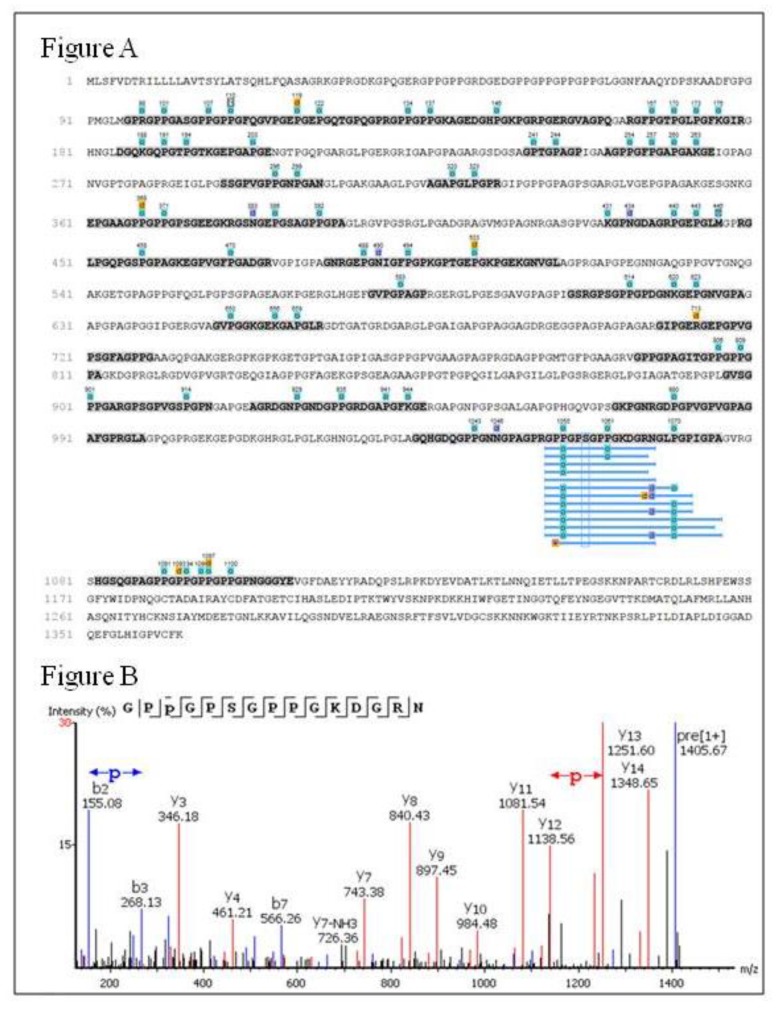
Identified post-translational modifications (PTM) in peptides from protein hydrolysis. (**A**) Assembly result for the identified peptide sequences from precursor protein Collagen alpha-2(I) chain in >DH. Gray lines indicate coverage regions from peptide spectrum matches and the tags indicate identified hydroxylation (blue), dihydroxy (yellow) and deamidation (purple). (**B**) MS/MS spectrum of the peptide GPP(+15.99)GPSGPPGKDGRN (*m/z* 703.34^+2^) showing hydroxylation in Proline^1055^ with *b* (blue) and *y* (red) ions matched.

A higher number of prolines present in chicken comb/wattles peptides were found with hydroxylation modification (hydroxyproline—Pro +15.99 Da) and some with motif Gly-Pro-Hyp like sequences GLP(+15.99)GPIGP(+15.99)P(+15.99)GPR and GPP(+15.99)GPSGPPGKDGRN (Figure 6). Protein hydrolysates produced from collagen-rich materials like skin gelatin have been reported to display antioxidant activity and particularly those having the repeating motif Gly-Pro-Hyp, found in the hydrolysates of the present chicken comb/wattles [12].

The oral ingestion of collagen peptides has been correlated with decreasing levels of platelet release indicators in plasma and increasing levels of type I collagen, which, in turn, improves aged skin collagen fibers [26]. This was particularly attributed to an enrichment of Pro-Hyp derived from Gly-Pro-Hyp hydrolysis, which was deposited in the skin [27]. In a clinical-laboratory study, the ingestion of collagen peptides containing Pro-Hyp increased skin elasticity, sebum production, dermal ultrasonic markers, plasma hydroxyproline level and adenosine triphosphate (ATP) storage in erythrocytes [28]. It is worth noting that all these bioactivities may vary from the present in vitro experiment to physiological conditions. In this regard, an upcoming study will cover the bioaccessibility of bioactive peptides from chicken combs and wattles after a simulated digestion model. 

## 3. Materials and Methods

### 3.1. Materials 

Chicken combs and wattles were acquired from a slaughterhouse located in the state of Paraíba (Brazil). The enzyme Alcalase (*Bacillus licheniformis*) was supplied by Novozymes Latino Americana Ltda. (Sao Paulo, Brazil). Other chemicals and reagents were obtained from Merk, (Sao Paulo, Brazil). 

### 3.2. Optimization of Hydrolysis of Extracts from the Chicken Comb + Wattle Mixture 

Chicken combs and wattles were ground and homogenized at the ratio of 1:1 (*w*/*w*). This mixture was transferred to a beaker in a pre-heated, temperature-controlled water bath. Ultra-pure water was added at a ratio of 1:2 (*w*/*v*), with constant stirring. Temperature (60 °C) and pH (8,0) were controlled according to the optimal performance values of the added enzyme. During hydrolysis, the pH was controlled by the addition of 0.5 mol/L NaOH. Alcalase was selected over other options (Flavouzyme from *Aspergillus oryzae*, Brauzyn from caricapapaya and Collagenase from *Clostridium histolyticum*), owing to the higher proteolytic activity of Alcalase on chicken combs and wattles in a preliminary study. 

The conditions (enzyme/substrate ratio and hydrolysis time) of protease-mediated hydrolysis of the chicken combs and wattles were optimized via a 2^2^ full factorial plan, which consisted of 4 factorial points and 3 central points for 7 experiments in total (Table 3). The independent variables investigated were X_1_ or E:S (enzyme/substrate ratio) and X_2_ or T (hydrolysis time in minutes). The response function was measured as the percentage of the degree of hydrolysis (% DH) [29].

The following model was used:

Y= β0 + β1(E:S) + β2(T) + β3(E:S)(T) + e,(2)

where Y is the response function predicted by the model, β_0_ is the average coefficient (or the constant), β_1_ and β_2_ are linear coefficients and β_3_ is the interaction coefficient. In this model, E:S and T are the independent variables, and the dependent variable is the degree of hydrolysis. After adjustment to the experimental data, the model (Equation 2) was verified using analysis of variance (ANOVA) and the coefficient of determination (R^2^). 

For validation of the model, a new test at the optimum point was performed in triplicate, and the results were compared via *t*-tests (*p* ≤ 0.05) with the response function estimated using the model.

### 3.3. Production of Enzymatic Hydrolysates

The enzyme to substrate ratio (5%) (*w*/*w*) and the total hydrolysis time (240 min) were determined according to the maximum levels to be used in the full factorial experimental plan aforementioned. After 240 min of hydrolysis, the enzyme was inactivated at 90 °C for 15 min. The hydrolysate was centrifuged (12,000× *g* for 30 min), and the supernatant was filtered and lyophilized to obtain a hydrolysate powder. 

### 3.4. Electrophoresis

The profiles of the protein and peptide fractions were determined using sodium dodecyl sulfate polyacrylamide gel electrophoresis (SDS-PAGE) to separate the higher molecular weight bands (225 to 38 kDa) [30] and using Tricine-SDS-PAGE to separate the lower molecular weight bands (38 to 3.5 kDa) [31].

### 3.5. Profile of Total and Free Amino Acids 

The total and free amino acids were hydrolyzed, extracted and derivatized with phenylisothiocyanate (PITC) according to the methods proposed by White, Hart and Fry [32] and Hagen, Frost and Augustin [33], respectively. Separation of the phenylthiocarbamyl amino acid derivatives was performed on a C18-reverse-phase (PICO-TAG, 3.9×150mm) high efficiency liquid chromatograph (Varian, Waters 2690, California, USA). The mobile phases employed consisted of an acetate buffer of pH 6.4 and a solution of 40% acetonitrile. Sample injection was performed manually (20 μL), and detection occurred at 254 nm. Separation of the amino acids was performed at a constant flow rate of 1 mL/minute at 35 °C. The chromatographic run time was 45 min. Quantification was performed using calibration curves of the separated and identified amino acids. The results were expressed as g of amino acid per 100 g of sample.

### 3.6. Fe^2+^ Chelating Capacity 

The Fe^2+^ chelating capacity was evaluated based on interruption of the formation of the F^2+^ —Ferrozine complex at 562 nm in a UV-VIS spectrophotometer (Quimis, São Paulo, Brazil) according to the method described by Stookey [34]. The percent inhibition of the ion complex formation (F^2+^–Ferrozine) was evaluated by a decrease in the color intensity, as greater inhibition indicates greater competition among peptides to form stable complexes with Fe^2+^ (thereby immobilizing Fe^2+^). The result was expressed as the percent inhibition of the ion complex.

### 3.7. Ferric Reducing Antioxidant Power (FRAP) 

The ferric reducing capacity was evaluated using the ferric reducing antioxidant power (FRAP) method described by Benzie and Strain [35]. According to the antioxidant potential, the ability of the hydrolysates to reduce iron (Fe^3+^) to the ferrous form (Fe^2+^) was verified at 593 nm in a UV-VIS spectrophotometer (Quimis, São Paulo, Brazil). Based on the calibration curve prepared with different concentrations of Trolox (1000 μmol/L), the results were expressed as the equivalent of mg of Trolox/100 g of sample.

### 3.8. Sequestering Activity for the 2,2-Diphenyl-1-Picrylhydrazyl Radical (DPPH^●^) 

The ability of the protein hydrolysates to sequester the DPPH^●^ radical was determined according to the method described by Brand-Williams, Cuvelier and Berset [36]. The radical elimination activity according to the antioxidant capacity of the hydrolysates was verified at 517 nm in a UV-VIS spectrophotometer (Quimis, São Paulo, Brazil) in triplicate. The antioxidant potential of the samples was expressed as the percent inhibition of the DPPH^●^ radical.

### 3.9. Sequestering Activity for the 2,2-Azino-bis (3-Ethylbeothiazoline)-6-Sulphonic Acid Radical (ABTS^●+^)

The ability to sequester the ABTS^●+^ radical was determined according to the method proposed by Re et al. [37]. The radical elimination activity was verified according to the antioxidant capacity of the hydrolysates at 734 nm in a UV-VIS spectrophotometer (Quimis, São Paulo, Brazil). The antioxidant potential of the samples was expressed as the percentage of inhibition of the ABTS^●+^ radical.

### 3.10. Identification of Peptide Sequences by Nanoflow Liquid Chromatography-Electrospray Ionization-Tandem Mass Spectrometry (nanoLC-ESI-MS/MS) Analysis

The peptide mixtures were subjected to nanoLC-ESI-MS/MS analysis performed on a Dionex Ultimate 3000 RSLCnano HPLC system (Thermo Scientific, Bremen, Germany) coupled with an Impact II quadrupole time-of-flight (Q-TOF) mass spectrometer. A volume of 2 µL was loaded for 8 min on an Acclaim Pepmap nano-trap column (Dionex-C18, 100 Å, 75 µm × 2 cm) and peptide mixture trapped was separated online using an Acclaim Pepmap RSLC analytical column (Dionex-C18, 100 Å, 75 µm × 15 cm) under gradient elution from 5% to 40% (*v*/*v*) of acetonitrile 0.1% FA for 120 min and flow rate set to 250 nL/min. Mass spectra were acquired in the positive-ion mode with MS precursors, and MS/MS products acquired at 2 Hz in the 50–3000 *m*/*z* mass range and ramped collision-induced dissociation (CID) energy parameters ranged from 7 to 70 eV. 

The raw MS data (d file) containing MS/MS spectra were imported in PEAKS Studio 8.5 software (Bioinformatics Solution Inc., Waterloo, Canada) for de novo analysis and database searches [38]. De novo analysis was carried out with a precursor mass tolerance of 10 ppm, fragment mass tolerance of 0.025 Da, no specific enzyme cleavage and was set as variable modifications oxidation on Met (+15.99 Da), dihydroxi on Tyr, Trp, Phe, Arg, Lys, Pro, Cys (+31.98 Da), oxidation or hydroxylation on Arg, Tyr, Pro, Asn, Lys, Asp (+15.99 Da) and deamidation on Asn, Gln (+0.98 Da).

De novo sequenced peptides with average local confidence (ALC) scores ≥ 50% were selected and submitted to database searches using SPIDER tools against the *Gallus gallus* database from UniprotKB/swiss-prot (2287 sequences) [39]. A false discovery rate (FDR) threshold of 1% on the peptide-spectrum match (PSM) was applied to filter out the peptide sequences. 

### 3.11. Statistical Analysis

The data obtained while optimizing the hydrolysis were evaluated using analysis of variance (ANOVA) with Statistica software, version 5.0 [40]. From these results, the effects of the studied variables were estimated, and the coefficients of the model for the experimental response were determined using a significance level of 5% (*p* ≤ 0.05).

The results of the protein hydrolysates with the highest and lowest degrees of hydrolysis were analyzed using analysis of variance (ANOVA) with Statistical Analysis System software, version 11.0 [41]. Significance was assigned at 1% and 5%.

## 4. Conclusions

By-products of chicken slaughter, combs and wattles, have been characterized as relevant sources of animal protein. The present study provides a feasible and optimized method for producing hydrolysates from these by-products using Alcalase. The hydrolysates display assorted bioactivities including Fe^2+^ chelating and antioxidant capacities. Bioactive peptides obtained from hydrolyzed proteins from chicken by-products are candidates to be used as functional ingredients in processed foods. Yet, the stability of these hydrolysates as well as their bioactivities under physiological conditions should be confirmed in further studies. 

## Figures and Tables

**Table 1:** Total and free amino acid profile (g/100 g sample) of the chicken comb and wattle mixture and the protein hydrolysates.

Amino Acids	Total Amino Acids			Free Amino Acids	
	C + W	<DH	>DH	<DH	>DH
Hydrophobic					
Glycine	9.05 ^b^ ± 0.02	11.76 ^a^ ± 0.09	8.82 ^c^ ± 0.00	0.76 ^b^ ± 0.01	1.37 ^a^ ± 0.01
Alanine	4.82 ^b^ ± 0.00	5.38 ^a^ ± 0.02	4.09 ^c^ ± 0.00	1.03 ^b^ ± 0.00	1.90 ^a^ ± 0.01
Leucine	2.88 ^c^ ± 0.00	3.60 ^a^ ± 0.02	2.95 ^b^ ± 0.00	1.25 ^b^ ± 0.01	2.02 ^a^ ± 0.01
Methionine	0.84 ^c^ ± 0.00	1.22 ^a^ ± 0.02	0.99 ^b^ ± 0.00	0.39 ^b^ ± 0.01	0.74 ^a^ ± 0.02
Isoleucine	0.32 ^c^ ± 0.00	2.09 ^a^ ± 0.00	1.74 ^b^ ± 0.00	0.56 ^b^ ± 0.01	0.95 ^a^ ± 0.01
Phenylalanine	0.90 ^c^ ± 0.03	1.80 ^a^ ± 0.01	1.49 ^b^ ± 0.00	0.65 ^b^ ± 0.00	1.02 ^a^ ± 0.01
Hydroxyproline	4.18 ^c^ ± 0.01	6.45 ^a^ ± 0.04	4.70 ^b^ ± 0.00	ND	ND
Proline	5.70 ^b^ ± 0.01	6.82 ^a^ ± 0.03	5.07 ^c^ ± 0.00	0.54 ^b^ ± 0.00	0.62 ^a^ ± 0.01
Serine	3.96 ^a^ ± 0.00	2.60 ^b^ ± 0.02	2.12 ^c^ ± 0.00	0.39 ^b^ ± 0.00	0.79 ^a^ ± 0.01
Tyrosine	0.69 ^c^ ± 0.00	1.17 ^a^ ± 0.00	0.99 ^b^ ± 0.00	0.33 ^b^ ± 0.00	0.74 ^a^ ± 0.00
Threonine	1.93 ^c^ ± 0.03	2.57 ^a^ ± 0.02	2.06 ^b^ ± 0.00	0.54 ^b^ ± 0.01	1.08 ^a^ ± 0.00
Valine	0.94 ^c^ ± 0.00	2.67 ^a^ ± 0.00	2.23 ^b^ ± 0.00	0.78 ^b^ ± 0.00	1.30 ^a^ ± 0.01
Tryptophan	ND	ND	ND	0.59^a^ ± 0.01	0.53^a^ ± 0.05
Total	36.20 ^c^ ± 0.00	48.12 ^a^ ± 0.02	37.2 4^b^ ± 0.00	7.78 ^b^ ± 0.00	13.03 ^a^ ± 0.02
Hydrophilic					
Aspartic acid	7.38 ^a^ ± 0.00	6.03 ^b^ ± 0.02	4.85 ^c^ ± 0.00	0.64 ^b^ ± 0.00	1.02 ^a^ ± 0.01
Glutamic acid	10.01 ^a^ ± 0.00	9.12 ^b^ ± 0.05	7.05 ^c^ ± 0.00	1.22 ^b^ ± 0.00	1.92 ^a^ ± 0.01
Arginine	4.37 ^c^ ± 0.00	5.53 ^a^ ± 0.02	4.55 ^b^ ± 0.00	0.63 ^b^ ± 0.01	1.93 ^a^ ± 0.01
Histidine	0.66 ^a^ ± 0.04	0.32 ^b^ ± 0.09	0.23 ^b^ ± 0.00	0.08 ^a^ ± 0.01	0.09 ^a^ ± 0.05
Lysine	1.95 ^c^ ± 0.01	3.56 ^a^ ± 0.03	2.80 ^b^ ± 0.00	0.88 ^b^ ± 0.01	1.34 ^a^ ± 0.01
Total	24.37 ^a^ ± 0.00	24.56 ^a^ ± 0.03	19.48 ^b^ ± 0.00	3.45 ^b^ ± 0.00	6.30 ^a^ ± 0.02

C + W, chicken comb and wattle mixture; <DH, protein hydrolysate of the chicken comb and wattle mixture with the lowest degree of hydrolysis; >DH, protein hydrolysate of the chicken comb and wattle mixture with the highest degree of hydrolysis. Different lowercase superscript letters indicate significant differences (*p* ≤ 0.01) between the samples. ND, not detected.

**Table 2:** Top 10 peptides most abundant in the different degrees of hydrolysis according to peak area under curve (AUC).

Peptide Sequences	Mass (Da)	Length	ppm	AUC
High Degree Hydrolysis				
GLP(+15.99)GPIGP(+15.99)P(+15.99)GPR	1161.61	12	−5.9	5.26 × 10^7^
GAP(+15.99)GDRGEP(+15.99)GPP(+15.99)GPA	1378.61	15	−7.7	2.94 × 10^7^
RGFP(+15.99)GLP(+15.99)GPS	1015.50	10	−7.2	2.14 × 10^7^
GDIGGP(+15.99)GFP(+15.99)GPK	1129.54	12	−9.1	2.05 × 10^7^
GAP(+15.99)GLP(+15.99)GPR	852.445	9	−9.6	1.59 × 10^7^
GKDGLNGLP(+15.99)GPIGP(+15.99)P(+15.99)GPR	1745.90	18	−6	1.31 × 10^7^
GPP(+31.99)GPPGPP(+15.99)GPP(+15.99)GPPSGGF	1685.76	19	−4.6	1.22 × 10^7^
RGPGGPP(+15.99)GTP(+15.99)GPA	1148.55	13	−7.9	1.18 × 10^7^
N(+.98)GDAGRP(+31.99)GEPGLM	1302.55	13	−7.1	1.16 × 10^7^
GEP(+15.99)GRSGPP(+15.99)GPAGPR	1419.68	15	−8.8	1.14 × 10^7^
Low Degree Hydrolysis				
TIGIGDIFDQPEQSETL	1861.89	17	−7	2.72 × 10^7^
GVDTKYVPPPFNPDMFSF	2056.96	18	−7.8	2.46 × 10^7^
GARGPAGPQGPRGDK(+15.99)GETGEQGDRGMK(+15.99)GH	2891.34	29	−9.5	1.24 × 10^7^
GDRGDP(+15.99)GPKGADGAPGKDGLRGL	2178.07	23	−8.9	7.89 × 10^6^
GLP(+15.99)GQPGSP(+31.99)GPAGKEGPVGFP(+15.99)GADGRVGPIGPA	3013.48	33	−7.4	7.23 × 10^6^
GPAGPQGPRGDK(+15.99)GETGEQGDRGMK(+15.99)GH	2607.18	26	−9.8	7.03 × 10^6^
GPAGNRGASGPVGAKGPNGDAGRP(+15.99)GEP(+15.99)GLMGPR	3042.48	33	−9.1	6.65 × 10^6^
GFP(+15.99)GADGIAGPKGPP(+15.99)GE	1554.73	17	−9.8	6.33 × 10^6^
P(+15.99)GADGIAGPKGPP(+31.99)GER(+31.99)	1554.72	16	−8.5	6.33 × 10^6^
GNRGEP(+15.99)GNIGFP(+15.99)GPKGPTGEPGKP(+31.99)GEKGNVGLAGPR	3515.75	36	−9.7	6.20 × 10^6^

Post-translational modifications: hydroxylation (+15.99 Da); dihydroxy (+31.98 Da).

**Table 3:** Full experimental plan involving 2^2^ + 3 repetitions of the central point.

Assay	Independent Variables and Variation Levels		Response Function (Y)
	Enzyme to Substrate Ratio (%)	Hydrolysis Time (minutes)	Degree of Hydrolysis (%)
1	−(1)	−(60)	7.51
2	+(5)	−(60)	13.55
3	−(1)	+(240)	12.19
4	+(5)	+(240)	19.34
5	0(3)	0(150)	14.90
6	0(3)	0(150)	14.29
7	0(3)	0(150)	14.53

Coded and real values of the experimental plan.

## References

[1] Borrajo P., Pateiro M., Barba F.J., Mora L., Franco D., Toldrá F., Lorenzo J.M. (2019). Antioxidant and Antimicrobial Activity of Peptides Extracted from Meat By-products: A Review. Food Anal. Meth..

[2] Toldrá F., Aristoy M.C., Mora L., Reig M. (2012). Innovations in value-addition of edible meat by-products. Meat Sci..

[3] Rosa C.S., Hoelzel S.C., Viera V.B., Barreto M., Beirão L.H. (2008). Atividade antioxidante do ácido hialurônico extraído da crista de frango. Ciênc Rural..

[4] Mora L., Toldrá F. (2012). Proteomic identification of small (<2000 Da) myoglobin peptides generated in dry-cured ham. Food Technol. Biotechnol..

[5] Mora L., Reig M., Toldrá F. (2014). Bioactive peptides generated from meat industry by-products. Food Res. Int..

[6] Bouacem K., Bouanane-Darenfed A., Laribi-Habchi H., Elhoul M.B., Hmida-Sayari A., Hacene H., Ollivier B., Fardeau M., Jaouadi B., Bejar S. (2015). Biochemical characterization of a detergent-stable serine alkalineprotease from Caldicoprobacter guelmensis. Int. J. Biol. Macromol..

[7] Choi J., Sabikhi L., Hassan A., Anand S. (2012). Bioactive peptides in dairy products International. Int. J. Dairy Technol..

[8] Lorenzo J.M., Munekata P.E.S., Gómez B., Barba F.J., Mora L., Pérez-Santaescolástica C., Toldrá F. (2018). Bioactive peptides as natural antioxidants in food products—A review. Trends Food Sci. Technol..

[9] Sarmadi B.H., Ismail A. (2010). Antioxidative peptides from food proteins: A review. Peptides.

[10] Acquah C., Di Stefano E., Udenigwe C.C. (2018). Role of hydrophobicity in food peptide functionality and bioactivity. J. Food Bioact..

[11] Ryan J.T., Ross R.P., Bolton D., Fitzgerald G.F., Stanton C. (2011). Bioactive peptides from muscle sources: Meat and fish. Nutrients.

[12] Zhou C., Li Y., Yu X., Yang H., Ma H., Yagoub A.E.A., Cheng Y., Hu J., Otu P.N.Y. (2016). Extraction and characterization of chicken feet soluble collagen. LWT Food Sci. Technol..

[13] Sae-Leaw T., Karnjanapratum S., O’Callaghan Y.C., O’Keeffe M.B., Fitzgerald R.J., O’Brien N.M., Bem S. (2017). Purification and identification of antioxidant peptides from gelatin hydrolysate of seabass skin. J. Food Biochem..

[14] Abdelhedi O., Jridi M., Jemil I., Mora L., Toldrá F., Aristoy M., Boualga A., Nasri M., Nasri R. (2016). Combined biocatalytic conversion of smooth hound viscera: Protein hydrolysates elaboration and assessment of their antioxidant, anti-ACE and antibacterial activities. Food Res. Int..

[15] Zhan P., Tian H., Zhang X., Wang L. (2013). Contribution to aroma characteristics of mutton process flavor from the enzymatic hydrolysate of sheep bone protein assessed by descriptive sensory analysis and gas chromatography olfactometry. J. Chromatogr. B.

[16] Chen N., Yang H., Sunc Y., Niu J., Liu S. (2012). Purification and identification of antioxidant peptides from walnut (*Juglans regia* L.) protein hydrolysates. Peptides.

[17] Zhang J.H., Zhang H., Wang L., Guo X.N., Wang X.G., Yao H.Y. (2010). Isolation and identification of antioxidative peptides from rice endosperm protein enzymatic hydrolysate by consecutive chromatography and MALDI-TOF/TOF MS/MS. Food Chem..

[18] Elango J., Jingyi Z., Wang N., He L., Bao B., Wu W. (2015). Physico-chemical, antioxidant and intestinal absorption properties ofwhale shark type-II collagen based on its solubility with acid and pepsin. Process Biochem..

[19] Alemán A., Giménez B., Pérez-Santin E., Gómez-Guillén M., Montero P. (2011). Contribution of Leu and Hyp residues to antioxidant and ACE-inhibitory activities of peptide sequences isolated from squid gelatin hydrolysate. Food Chem..

[20] Farvin K.H.S., Andersen L.A., Otte J., Nielsen H.H., Jessen F., Jacobsen C. (2016). Antioxidant activity of cod (*Gadus morhua*) protein hydrolysates: Fractionation and characterisation of peptide fractions. Food Chem..

[21] Ngoh Y., Gan C. (2016). Enzyme-assisted extraction and identification of antioxidative and α-amylase inhibitory peptides from Pinto beans (*Phaseolus vulgaris* cv. Pinto). Food Chem..

[22] Zou T., He T., Li H., Tang H., Xia E. (2016). The Structure-Activity Relationship of the Antioxidant Peptides from Natural Proteins. Molecules.

[23] Samaranayaka A.G.P., Li-Chan E.C.Y. (2011). Food-derived peptidic antioxidants: A review of their production, assessment, and potential applications. J. Funct. Foods.

[24] Shazly A.B., He Z., El-Aziz M.A., Zeng M., Zhang S., Qin F., Chen J. (2017). Fractionation and identification of novel antioxidant peptides from buffalo and bovine casein hydrolysates. Food Chem..

[25] Sun N., Cui P., Jin J., Wu H., Wang Y., Lin S. (2017). Contributions of molecular size, charge distribution, and specific amino acids to the iron-binding capacity of sea cucumber (*Stichopus japonicus*) ovum hydrolysates. Food Chem..

[26] Song H., Zhang L., Luo Y., Zhang S., Li B. (2018). Effects of collagen peptides intake on skin ageing and platelet release in chronologically aged mice revealed by cytokine array analysis. J. Cell. Mol. Med..

[27] Yazaki M., Ito Y., Yamada M., Goulas S., Teramoto S., Nakaya M., Ohno S., Yamaguchi K. (2017). Oral Ingestion of Collagen Hydrolysate Leads to the Transportation of Highly Concentrated Gly-Pro-Hyp and Its Hydrolyzed Form of Pro-Hyp into the Bloodstream and Skin. J. Agric. Food Chem..

[28] De Luca C., Mikhal’chik E.V., Suprun M.V., Papacharalambous M., Truhanov A.I., Korkina L.G. (2016). Skin Antiageing and Systemic Redox Effects of Supplementation with Marine Collagen Peptides and Plant-Derived Antioxidants: A Single-Blind Case-Control Clinical Study. Oxid. Med. Cell. Longev..

[29] Adler-Nissen J. (1986). Enzymic Hydrolysis of Food Proteins.

[30] Laemmli U.K. (1970). Cleavage of structural proteins during the assembly of the head of bacteriophage T4. Nature.

[31] Schagger H., Joagow V. (1987). Tricine-sodium dodecyl sufate-polyacrylamide gel electrophoresis for the separation of proteins in the range from 1 to 100 kDa. Anal. Biochem..

[32] White J.A., Hart R.J., Fry J.C. (1986). An evaluation of the Waters Pico-Tag system for the amino-acid analysis of food materials. J. Anal. Methods Chem..

[33] Hagen S.R., Frost B., Augustin J. (1989). Pre column phenylisothiocyanate derivatization and liquid chromatography of amino acids in food. J. Assoc. Off. Anal. Chem..

[34] Stookey L.L. (1970). Ferrozine-a new spectrophotometric reagent for iron. Anal. Chem..

[35] Benzie I.F.F., Strain J.J. (1999). Ferric reducing/antioxidant power assay: Direct measure of total antioxidant activity of biological fluids and modified version for simultaneous measurement of total antioxidant power and ascorbic acid concentration. Methods Enzymol..

[36] Brand-Wiliams W., Cuvelier M.E., Berset C. (1995). Use of a free radical method to evaluate antioxidant activity. Food Sci. Technol..

[37] Re R., Pellegrini N., Proteggente A., Pannala A., Yang M., Rice-Evans C. (1999). Antioxidant activity applying an improved ABTS radical cation decolorization assay. Free Radic. Biol. Med..

[38] Zhang J., Xin L., Shan B., Chen W., Xie M., Yuen D., Zhang W., Zhang Z., Lajoie G.A., Ma B. (2012). PEAKS DB: De Novo sequencing assisted database search for sensitive and accurate peptide identification. Mol. Cell. Proteomics.

[39] UNIPROT. http://www.uniprot.org/.

[40] Statsoft Inc (2004). Statistica (Data Analysis Software System).

[41] SAS Institute (2014). SAS User's Guide: Statistics.

